# Diseases of the Heart and Circulation

**Published:** 1904-01-09

**Authors:** 


### Diseases of the Heart and Circulation.

**Variations in Blood-Pressure.**—Oliver[1] gives an account of the physiological variations in the blood-pressure, as determined by the sphygmometer and hæmodynometer. There is a wave-like rise and fall in the arterial pressure produced by each meal lasting from two-and-a-half to four hours. Thus a normal wave rises from 100 to 115 mm. of mercury. During digestion there is dilatation of the splanchnic arterioles, a compensatory reduction of the systemic area of the circulation owing to increased tonus of the arterioles, and an increased output from the heart. The capillary blood-pressure is also considerably raised, whilst the venous pressure falls. The rise in the capillary pressure causes an increased exudation of tissue fluid. When food is taken in there is thus not only a flow of digestive secretions but also a concurrent exudation into every tissue of nourishing fluid. The primary effect of exercise is to raise the arterial pressure, but this is only temporary, and it is soon succeeded by a marked fall which continues during the exercise and for some time afterwards. There is thus a large volume of arterial blood in the capillaries, and an increased supply of oxygen to the tissues. In some cases of arterial hypertonus both the capillary and venous pressures are lowered together showing the predominence of arteriolar contraction, and in these cases the hands and feet are apt to be cold and clammy, and the pulse is small. But the heart is hypertrophied and the second aortic sound accentuated. The trouble is really peripheral, and the treatment should be directed to produce dilatation of the arterioles. Tension or static muscular exercises are of great value, and patients find they make the extremities warm. Such exercises are much more beneficial than the application of warmth. The application of external cold, *e.g*, a cold atmosphere, increases the arteriole contraction and raises the blood pressure, thus putting some strain on the heart. Patients suffering from arterial hypertonus need a warm winter climate as much as a bronchitic individual. Altitude—up to about 3,000 feet—lowers the blood pressure, above that height the pressure rises and the pulse is slightly quicker. This affords an explanation of the facts that great heights are bad for patients with arterial hypertonus, poor hearts or Bright's disease.

**Angina Pectoris.**—Colbeck has put forward a theory in explanation of the symptoms of angina pectoris. He first observes that the theory of muscular cramp does not explain the phenomena, for, in the first place, there is no physiological evidence that the cardiac muscle can pass into a state of cramp like the skeletal muscles; again, even if it could take place it would not account for the fact that during the attack the cardiac action still goes on. His theory depends on the well-known fact that disease of the coronary arteries and of the myocardium is in some way associated causally with the incidence of angina pectoris. The characteristic of these myocardial changes is that they are irregularly distributed over the substance of the cardiac walls. These degenerated areas are unable to take their proper share in resisting the increased intra-cardiac pressure, and in consequence undergo more or less distension and stretching. The pain experienced is due to this. The ventricular systole takes place as usual. Hence, as is well known, the action of the heart and the rhythm and character of the heart may remain, as far as physical examination is concerned, undisturbed during the paroxysm. The establishment of mitral regurgitation relieves the pain by lessening the intra-ventricular pressure, and vaso-dilators act similarly. The sense of impending death—the angor animi—is explained thus: if distension of localised patches in the ventricular wall takes place during systole, these areas are simultaneously undergoing contraction and expansion, and send antagonistic impulses to the cardiac centre which is thus thrown into a turmoil. The perturbation of the cardiac spreads to adjacent centres, and herein lies the explanation of the vaso-motor phenomena as well as of the nausea, vomiting, flatulence and hiccough, which so frequently accompany the paroxysm. Angina sine dolore is explained on the assumption that the deterioration of the nervous elements in the cardiac wall has become so great that they fail to react to stimuli which ordinarily give rise to the sensation of pain.

**Infective Endocarditis.**—In the Lumleian lectures of 1903 the clinical aspects of infective endocarditis were discussed by Glynn.[3] The recent discovery that the specific microbe of rheumatism may excite endocarditis in its fungating as well as in its simple form, leads to a division of malignant endocarditis into a rheumatic form, and a septic form occasioned by the pyococci. Since both forms of endocarditis, the "simple" and the "malignant," are occasioned by microbes the terms non-infective and infective are hardly justifiable; and the distinction between simple and malignant is merely one of degree. In simple endocarditis the lesions are localised and tend to develop into fibrous tissue, the infective character being gradually lost. In the malignant form, the lesions preserve their infective character and tend to diffuse themselves. Malignant endocarditis is prone to attack the debilitated, cachectic and intemperate, it may follow anæmia, parturition and pneumonia and rheumatism; and it is very frequently associated with sclerotic changes in the endocardium. The vegetations are usually of considerable size, and at times form fungating masses. When recent they are soft, friable, and easily detached, and generally pale pink in colour. Embolism is nearly three times as common in malignant as in simple endocarditis; suppuration of the infarcts rarely occurs. Any attempt to classify the varieties of endocarditis on an anatomical basis, as into ulcerative and verrucose forms, is not justified, for the two kinds of lesion very frequently coexist; nor is a classification founded on bacteriological investigation of much clinical value, for the severity and character of the symptoms depends on the virulence rather than on the species of the infecting micro-organisms. It has not been established that the infection produced by the various kinds of bacteria produce distinguishing clinical differences. Pneumococcus endocarditis more often produces meningitis than does infection by other micro-organisms. A clinical basis is the best one for any classification, and cases may be divided into acute, subacute and chronic forms. More or less pyrexia is usually a constant feature though sometimes it is conspicuous by its absence. The character of the temperature affords some indication of the type and severity of the disease; it generally assumes a continuous, intermittent, or irregular intermittent form. In very few patients were there rigors. Progressive anæmia is a marked characteristic. Epistaxis and petechiæ may occur. Gastro-intestinal symptoms are common—especially diarrhœa and vomiting. Albuminuria and hæmaturia are common—sometimes the result of infarctions and sometimes of inflammatory changes. Enlargement of the spleen is almost invariably present, and is a valuable physical sign. When embolism of the spleen occurs in a patient under observation and is attended with pain, swelling, and the development of friction sounds over the organ, or when embolism of a kidney takes place and gives rise to hæmaturia, valuable indications of the nature of an endocarditis are afforded. The endocardial murmurs in many of the cases are not due to the recent endocarditis nor even modified by it, but are the consequence of the chronic lesions; in some cases the bruits may, as the structures become more deteriorated, suddenly undergo great alterations in character. When the affection commences insidiously without initiatory rigors, as is frequently the case in chronic forms, it is difficult to ascertain the date of the invasion, but in some cases some idea may be obtained from the history of night sweats and diarrhœa. The cause of death may be cerebral embolism or hæmorrhage, pneumonia, cachexia, due to the persisting septicæmia, or uræmia. The difficulties of diagnosis are often considerable and sometimes insuperable. For instance, a man, aged 51, was admitted to hospital with albuminuria, irregular pyrexia, evidence of aortic and mitral disease and enlargement of the spleen. At the necropsy the aorta was found to be atheromatous and its valves were incompetent, but there was no recent endocarditis. The pyrexia was due to miliary tubercles in the lungs. Again, when phthisis is associated with chronic valvular disease the resultant symptoms necessarily conform to the chronic type of infective endocarditis. The protracted forms of infective endocarditis present a close resemblance to pernicious anæmia, and the likeness is the closer since streptococci may be found in the blood in both diseases. The occurrence of leucocytosis may help to distinguish infective endocarditis from typhoid fever. The disease is generally fatal, though occasionally it may end favourably.

[1] Lancet, June 13. [2] Lancet, March 21. [3] Lancet, April 11, 18, and 25.

*(To be continued.)*

